# Diet composition analysis provides new management insights for a highly specialized endangered small mammal

**DOI:** 10.1371/journal.pone.0240136

**Published:** 2020-10-02

**Authors:** Stephanie T. Castle, Nora Allan, Deana Clifford, Cody M. Aylward, Jon Ramsey, Andrea J. Fascetti, Risa Pesapane, Austin Roy, Mark Statham, Benjamin Sacks, Janet Foley

**Affiliations:** 1 Department of Medicine and Epidemiology, School of Veterinary Medicine, University of California, Davis, CA, United States of America; 2 Wildlife Investigations Lab, California Department of Fish and Wildlife, Rancho Cordova, CA, United States of America; 3 Department of Fish, Wildlife and Conservation Biology, University of California, Davis, CA, United States of America; 4 Mammalian Ecology and Conservation Unit, Veterinary Genetics Laboratory, University of California, Davis, CA, United States of America; 5 Department of Molecular Biosciences, School of Veterinary Medicine, University of California, Davis, CA, United States of America; 6 Department of Population Health, and Reproduction, School of Veterinary Medicine, University of California, Davis, CA, United States of America; Sichuan University, CHINA

## Abstract

The critically endangered Amargosa vole (*Microtus californicus scirpensis*) is found only in rare marsh habitat near Tecopa, California in a plant community dominated by three-square bulrush (*Schoenoplectus americanus*). Since the earliest research on the Amargosa vole, the existing paradigm has been that these voles are obligatorily dependent on bulrush as their only food source and for the three-dimensional canopy and litter structure it provides for predator avoidance. However, no prior research has confirmed the diet of the Amargosa vole. In this study we characterized the Amargosa vole’ nutritional needs, analyzed the quality of bulrush by forage analysis, and performed microhistological and metabarcoding analyses of vole feces to determine what foods were consumed in the wild. All bulrush plant tissues analyzed were low in fat (from 0.9% of dry matter in roots to 3.6% in seeds), high in neutral detergent fiber (from 5.9% in rhizomes to 33.6% in seeds), and low in protein (7.3–8.4%). These findings support the conclusion that bulrush alone is unlikely to support vole survival and reproduction. Fecal microhistology and DNA metabarcoding revealed relatively diverse diets including plants in 14 families, with rushes (Juncaceae), bulrushes (Cyperaceae), and grasses (Poaceae) being the most common diet items. On microhistology, all analyzed samples contained bulrush, sedges (*Carex* sp.), rushes (*Juncus* sp.), and beaked spikerush (*Eleocharis rostrellata*) even from marshes where non-bulrush plants were uncommon. There was evidence of insects at <1% in two marshes but none in the remaining marshes. Metabarcoding detected ten genera of plants. When considering non-*Schoenoplectus* targets, for which metabarcoding had poor sensitivity, saltgrass (*Distichlis spicata*) was the most commonly detected species, with prominent contributions from seaside arrowgrass (*Triglochin concinna*) and yerba mansa (*Anemopsis californica*) as well. Diversity of vole diets generally increased with increasing site plant diversity, but differences were not statistically significant. Confirming details about dietary behaviors is critical for informing appropriate conservation planning including habitat management and reintroduction of voles into new sites.

## Introduction

Climate change, habitat loss, and other human-mediated disturbances are threats to endangered species world-wide, particularly those with highly specialized habitat and diet requirements [1, 2]. Reduced availability and dependability of nutritional resources can impact survivorship, reproduction, population size, and species’ probabilities of persistence [3–6]. Accurate and detailed information about the dietary needs of endangered species is crucial for endangered species management [7, 8].

The habitat-specialist Amargosa vole (*Microtus californicus scirpensis*) is one of the most endangered mammals in North America [9]. It occupies a footprint of less than 40 ha of rare and deteriorating marsh habitat in the lower Amargosa River basin in the Mojave Desert near Tecopa, California [9, 10]. Marshes inhabited by Amargosa voles are dominated by relative monocultures of Olney’s three-squared bulrush (*Schoenoplectus americanus*), which provides forage, structural protection from predators, and thermal refugia in an location characterized by wide fluctuations in ambient temperature [11–13]. Since the original description of the species in the late 19^th^ century [14], obligate dependence of voles on bulrush for nutrients has been explicitly reported or assumed [12, 15, 16]. However, bulrush is very low in fat and protein, rendering it unclear how a rodent could subsist entirely on such a nutrient-poor food source.

There is evidence that the diet of Amargosa voles may be broader than bulrush. California voles, of which the Amargosa vole is a subspecies, are widely distributed across a range of habitats in western North America, from grasslands to arid uplands. Most California voles feed on grasses, forbs, and sedges and tend to rely on seeds and roots during summer months [10, 17]. When being introduced to captivity, individual Amargosa voles showed considerable reluctance to eat including when offered bulrush, but eventually accepted jicama and sweet potato [18]. On two occasions, remotely-triggered cameras deployed in Tecopa marshes captured observations of Amargosa voles feeding on non-bulrush plants, including yerba mansa (*Anemopsis californica*) and clustered goldenweed (*Pyrrocoma racemosa*, Roy, unpub. data). In contrast, when voles in a captive colony were gradually restricted to a bulrush-only diet, animals were unable to maintain body mass and condition [18].

To date, no studies have confirmed what constitutes the diet of the Amargosa vole. In this study, we performed nutritional analysis of bulrush from marshes inhabited by Amargosa voles and compared these results to the calculated expected dietary requirements of voles. Diet composition of wild Amargosa voles was analyzed using fecal microhistology and DNA metabarcoding analysis to determine whether the voles’ use of bulrush or other plant species was influenced by plant species diversity within their home range. Such information may enhance success of management programs for the vole, including captive breeding and habitat rehabilitation.

## Materials and methods

### Study site

This study was conducted in marshes of the lower Amargosa River basin, near Tecopa, California in the Mojave Desert (35^o^52’20” N, 116 ^o^ 13’57” W). Average daytime temperatures ranging from 15°C to 23°C during winter months and 37°C to 43°C during summer months. Mean annual precipitation (1972–2018) is 120 mm, with approximately 70% of precipitation occurring from November through March [19]. The marshes form disjunct patches within a matrix of desert landscape that is otherwise inhospitable for voles. Predominant plant species in marshes occupied by voles are bulrush and desert saltgrass (*Distichlis spicata*) with patches of rushes (*Juncus cooperi* and *J*. *balticus*), yerba mansa, and boraxweed (*Nitrophila occidentalis*) along marsh edges.

### Bulrush nutritional analysis

Live native root, rhizome, stem, and leaf tissues of bulrush were collected in July 2015 and pooled in approximately equal mass by tissue type from marshes within the vole’s range in Tecopa, California. Root, rhizome, stem, leaves, and flower heads with seeds were also sampled from a clone of Tecopa bulrush propagated in a greenhouse at the University of California, Davis. Plant tissues were thoroughly rinsed with deionized water to remove mineral and soil residues and then sent to Dairy One Forage Laboratory (Ithaca, NY) for wet chemistry analysis of dry matter, crude protein, acid-detergent fiber, neutral-detergent fiber, non-fiber carbohydrates, and total digestible nutrients.

### Calculation of Amargosa vole dietary needs

Metabolic and nutritional data for the closely related California vole [10, 20, 21] were used to estimate daily energy (calories/g body mass/day) and nutritional requirements (% of key nutrient) for Amargosa voles using a simple mass balance approach.

### Fecal sample collection

All activities were performed in accordance with guidelines or permits from the American Society of Mammologists [22], US Fish and Wildlife Service Amargosa vole Recovery Permit #TE546414A-2, California Department of Fish and Wildlife Scientific Collecting Permit #854, and the UC Davis Institutional Animal Care and Use Committee. Voles were live-trapped as described previously for studies of population status and disease, as well as to create a captive-breeding colony at the University of California, Davis (UCD) [12, 18, 23].

At each sampling location, GPS coordinates and all plant species within 50 m were recorded. Sherman live traps (HB Sherman, Tallahassee, FL) were placed deep in bulrush litter and baited with peanut butter and oats. Traps were set overnight and checked in the early morning before the heat of the day. Voles were processed to collect samples, for health checks, and to place ear tags. Fresh samples were also collected directly from litter. During work in Tecopa in April 2018, fecal pellets for microhistology were sampled from litter in three to four locations per marsh in five different marshes. All pellets from each marsh were pooled to form one sample for microhistology for that marsh. Vole fecal pellets were also collected in May 2019 for metabarcoding from vole-occupied live traps and directly from litter in vole-occupied marshes. Approximately 4–6 fecal pellets were collected from each trap or marsh site, including samples from 13 voles, 2 samples deposited on top of traps, and 17 samples collected from bulrush litter, and stored in microtubes with 100% ethanol.

### Microhistological analysis of feces

The exteriors of samples were scraped to remove any algal or plant litter residues. Pellets that were severely decomposed, moldy, or had extensive attached plant residues attached were discarded. Remaining pellets were dried at 60°C for 24 hours. Dried samples were shipped to the Wildlife Habitat and Nutrition Laboratory (Pullman, WA) for microhistological diet composition analysis using their previously reported techniques [24]. Oven-dried samples were agitated with a blender and then washed over a 75 μm mesh screen. They were stained, mounted on a slide, and then examined under light microscopy at 100x with a gridded eyepiece. Relative cover [25, 26] of plant cuticle and epidermal fragments were quantified in 100 randomly chosen fields of view (25 views per slide, 4 slides per sample). Percent diet composition was calculated by dividing cover of each plant by total cover observed for all species, and then multiplying by 100.

### DNA metabarcoding of feces

DNA was extracted from Amargosa vole fecal pellets and exemplars of *Schoenoplectus*, *Triglochin*, and *Juncus* using Plant Mini Kits (Qiagen, Valencia, CA). DNA libraries were prepared using a modified Illumina 16S metagenomics protocol [27]. The internal transcribed spacer 2 (ITS2) region of nuclear DNA was amplified via polymerase chain reaction (PCR) using universal primers UniPlantF [28] and ITS-p4 [29], modified with additional sequence to allow annealing to Illumina indexed adapters. The final UniPlantF primer sequence was: TCTTTCCCTACACGACGCTCTTCCGATCTGTGAATTGCARRATYCMG, and ITS-p4 was: GTGACTGGAGTTCAGACGTGTGCTCTTCCGATCCCGCTTAKTGATATGCTTAAA. PCR mixes of 23 μL total volume included 2 μL template DNA, 1X PCR buffer, 2.5 mM MgCl, 0.2 mM dNTPs, 0.1 μg/μL BSA, 0.5 μM each primer, and 1 U Taq. The PCR thermal profile was 94°C for 4 min, 34 cycles of 94°C for 30 sec, 55°C for 40 sec, and 72°C for 60 sec, and a final extension at 72°C for 10 minutes.

PCR clean up, indexing, and library quantification and normalization were conducted according to the Illumina metagenomics protocol. The pooled library was sequenced on a MiSeq PE 300 at the UC Davis Genome Center. A custom reference sequence database was downloaded from BOLD and NCBI databases using PrimerMiner (Elbrecht and Leese 2017). The batch_download function was performed using marker queries “ITS2” and “internal transcribed spacer 2”. Next, sequence files for each taxon were individually formatted to Dada2 [30] reference data format and concatenated into a single reference database file.

Fastqc [31] and Multiqc [32] were used to visualize quality of sequence reads. Primer and adapter sequences were removed and reads shorter than 100 bp or <30 quality score were filtered out using Cutadapt [33]. Dada2 was used to estimate sequencing error rates and determine unique amplicon sequence variants (ASVs). ASVs were assigned to potential dietary items at the genus level from the custom reference sequence database. We used the assignTaxonomy function with a threshold of 95% bootstrap support for ASV taxonomy assignment in Dada2. The number of sequence reads of each genus was tabulated and converted to proportions after removing any taxon present in < 0.3% of a sample’s sequence reads to minimize false-positive detections due to sequencing errors. Reads corresponding to plant taxa present in bait (oats, *Avena*, and peanuts, *Arachis*) were also removed. The frequency of occurrence for each dietary item was calculated as the number of samples in which the item was present divided by the total number of samples. The relative read abundance (RRA) of each dietary item was calculated for each individual by dividing the number of reads of each dietary item by the total number of reads in the sample. RRAs were standardized such that, after removal of all non-diet reads (e.g., non-plant, rare items, or bait reads), RRA summed to 1 for each individual. All data are accessioned in NCBI under BioProject PRJNA648955.

### Data analysis

Data were managed in Excel (Microsoft, Redmond, WA) and analyzed using “R” [34]. Marshes were classified for plant species richness using data collected in earlier surveys [13] as high diversity (> 6 species present) or low diversity (≤ 5 plant species present, Table 1). Importantly, the flora of low diversity marshes was composed to a very great degree of bulrush. Microhistology results between three marshes with high plant species diversity and two marshes with low plant species diversity were compared, evaluating whether the species richness of diet items in feces and percent of diet containing bulrush differed by marsh species diversity using Student’s t-tests. Metabarcode RRA was summarized by marsh and by marsh diversity status, after summing RRA of all genera for each individual and dividing each genus’ RRA by the total for that marsh. As for microhistology, we compared diet species richness in high and low diversity marshes with a Student’s t-test.

**Table 1:** Plant species present in marshes (designated by M-number) reported in [13], sampled for vole feces to detect diet items.

Plant Species	Plant Family	M1	M54	M6	M7	M9	M10	M21	M39
*Schoenoplectus americanus*, three-square bulrush	Cyperaceae	X	X	X	X	X	X	X	X
*Carex* sp., sedge	Cyperaceae						X		
*Eleocharis rostellata*, beaked spikerush	Cyperaceae					X	X		
*Juncus cooperi*, Cooper’s rush	Juncaceae	X	X	X	X	X	X	X	X
*Juncus balticus*, Baltic rush	Juncaceae			X	X	X	X	X	X
*Triglochan concinna*, seaside arrowgrass	Juncagin-aceae		X	X	X	X	X	X	X
*Distichlis spicata*, desert saltgrass	Poaceae	X	X	X	X	X	X	X	X
*Sporobolus airoides*, alkali sacaton	Poaceae			X	X	X	X	X	X
*Phragmites australis*, common reed	Poaceae					X			
*Pyrrocoma racemosa*, clustered goldenweed	Asteraceae			X	X	X	X	X	X
*Helianthus annuus*, common sunflower	Asteraceae					X			X
*Almutaster pauciflorus*, alkali marsh aster	Asteraceae							X	X
*Anemopsis californica*, yerba mansa	Saururaceae			X	X	X	X	X	X
*Nitrophila occidentalis*, boraxweed	Amaranth-aceae	X		X	X	X	X	X	X
*Chloropyron tecopense*, Tecopa bird’s-beak	Orobanch-aceae			X	X	X	X	X	
*Typha domingensis*	Typhaceae	X							
Total Richness		5	4	10	10	13	12	10	11

## Results

### Nutritional analysis of bulrush forage and calculation of Amargosa vole dietary needs

Maintenance energy requirements for the California vole were estimated to be 0.661 Kcal/g digestible energy body mass/day [10]. Given that the mean mass of a wild adult, Amargosa vole is 80g, range 50g-110g [35], the daily energy requirements of an Amargosa vole were estimated to be 52.88 Kcal/day. From literature sources on dietary needs of captive and wild voles [10, 20, 21], it was estimated that 11–13% of the 52,880 daily calories should be from protein.

Nutritional analyses revealed that all bulrush tissues are low in fat (from 0.9% in rhizomes to 3.6% in seeds) and high in neutral detergent fiber (from 5.9% in rhizomes to 33.6% in seeds) (Table 2). Bulrush seeds have a low water content compared with other plant tissues, and roots contain the least amount of digestible energy (2.17 Kcal/g). Leaves and rhizomes have similar concentrations of digestible energy (2.48 Kcal/g and 2.43 Kcal/g respectively). The seeds are the most nutrient-dense component of the plant (2.59 Kcal/g). To meet their daily caloric requirements solely on a bulrush diet, Amargosa voles would need to consume a minimum of 33g of seeds, 77g leaves, or 189 g of rhizomes/day (calculations performed using a mass balance as shown in Appendix I). Protein concentrations of all plant tissues ranged from 7.3–8.4%, below the minimum daily protein requirement for voles.

**Table 2:** Estimated dietary needs of Amargosa voles and nutritional content of bulrush (dry matter).

	Nutritional needs of the Vole	Bulrush leaves	Seeds	Rhizomes	Roots
Digestible energy (cal/g)	661	2430	2590	2490	2170
Metabolizable energy (cal/g)	NA	2000	2170	2060	1740
Net energy for maintenance (cal/g)	NA	1140	1270	1190	940
Dry Matter (%)	NA	28.3	61.7	11.6	8.1
Total digestible nutrients (TDN, %)	NA	56	59	57	49
Acid digestible fiber (ADF, %)	NA	49.8	34.1	31.3	57.6
Neutral digestible fiber (%)	NA	61.8	54.4	51.4	80.7
Fat (%)	NA	2.3	3.6	1.3	0.9
Protein (%)	11–13	8.0	8.4	7.3	7.7
Calcium (%)	0.3–0.5	0.49	0.18	0.30	0.66
Phosphorus (%)	0.2–0.4	0.08	0.21	0.15	0.07
Sodium (%)	0.02	0.56	0.024	0.62	0.67

### Fecal microhistology analysis

Microhistological analysis of fecal pellets revealed a more diverse diet than reported previously. Fecal pellets contained five to eight different plant species (Table 3). Bulrush was included in all diet samples and was the dominant component in four of the five marshes sampled, comprising 48.8–77.7% of the vole diet (Table 2). Rushes were also present in all samples, as were sedges (*Carex* sp.) and beaked spikerush (*Eleocharis rostellata*), even though these latter taxa were not found in two of the marshes (M1 and M54). The primary contributions to the sample in which bulrush was not predominant were 51.3% sedge, 38.8% rushes, and 7% bulrush. Leaves were the most common plant tissue (95.8–99.7%) in all samples, with only 0.3–3.5% seeds and 0–0.8% roots. Samples from two marshes contained insects at less than 1%.

**Table 3:** Percentage of microscopy fields containing each plant species or tissue of pooled Amargosa vole fecal samples from five Tecopa, CA marshes examined with microhistology.

Plant Species	Marsh 1	Marsh 54	Marsh 6	Marsh 10	Marsh 21
*Distichlis spicata*	7.5	5	7.4	0	13.2
*Sporobolus airoides*	0	0	2.1	0	1.1
Unknown Grasses	0	0	1.1	0	2.2
Total Grasses:	7.5%	5.0%	10.6%	0.0%	16.5%
*Schoenoplectus americanus*	77.7	62.3	48.8	7	52.7
*Carex* sp.	2.9*	8.8*	7.4	51.3	8.4
*Juncus* spp.	8.9	9.3	12	38.8	14.6
*Eleocharis rostellata*	2.3*	8.8*	5.3	1.1	6.2
Total Sedge/Rush:	91.8%	89.2%	73.5%	98.2%	81.9%
*Triglochin concinna*	0	5.5	11.7	0	0.5
*Chloropyron tecopense*	0	0	0	0.7	0
Total Forbs/Herbs:	0%	5.5%	11.7%	0.7%	0.5%
Total number plant species	5	6	8	5	8
Total Root	0%	0%	0%	0%	0.8%
Total Seed	0.7%	0.3%	3.5%	0.4%	0.3%
Total Insect	0%	0%	0.7%	0.7%	0%

Marshes 1 and 54 had low plant diversity (≤ 4 plant species present); remaining marshes had high plant diversity (>6 plant species present). * denotes plants found in fecal diet analysis that were not detected in the marsh from which the sample was obtained.

Plant diversity in each marsh did not necessarily limit the number of species identified in fecal samples. Low-diversity each contained four plant species. However, fecal samples from those same sites contained five and six plant species (mean 5.5 ± 0.7 SD), respectively. The mean species richness in high-diversity marshes was 10.7 ± 1.6 SD. There was a mean number of seven plant species ± 1.7 SD in fecal samples. Differences in mean plant species represented in feces from low- and high-diversity marshes were not significant (P = 0.17). Mean percent bulrush in fecal samples from low diversity marshes was 70.0% ± 10.1 SD, compared with 36.2% ± 25.3 SD in high-diversity marshes; these differences also were not significant (P = 0.09).

### Fecal metabarcoding analysis

After quality filtering, 1.6 million sequence reads were obtained. Approximately 49% (783,042 reads) were assigned to plant taxa from the reference database. After removing reads from positive controls and bait (*Avena*, *Arachis*), there were 96,203 reads that likely originated from dietary items. After data curation, metabarcoding analysis with *ITS2* identified diet items from 10 plant genera belonging to 8 plant families including *Acmispon* or *Lotus*, ragweed (*Ambrosia* sp.), yerba mansa, saltgrass, *Gilia*, sunflower (*Helianthus annuus*), bulrush, clover (*Trifolium* sp.), and seaside arrowgrass (*Triglochin concinna*). There was only one marsh (Marsh 7) from which samples were available from the trap and the environment: trap samples had *Avena* from bait 97% of the plant reads in the sample from the trap) and DNA of yerba mansa, compared with environmental samples from Marsh 7 which included yerba mansa (non-zero RRA from 0.02–1.00, depending on the sample), ragweed (0.03–1.00), saltgrass (0.87–0.95), and bulrush (0.01).

Positive control samples suggested low sensitivity for detection of bulrush. Only 16% of reads in a sample of bulrush DNA were identified as bulrush, and in two mixtures that contained 50% bulrush DNA, only 8% and 5% of reads, respectively, were identified as bulrush. As microhistology results had confirmed bulrush as a common diet item and metabarcoding controls suggested a high chance of false-negatives, bulrush was excluded from further plots and statistical tests, restricting comparison to analysis of non-bulrush diet items except for the comparison of species richness in diet.

**Fig 1:**
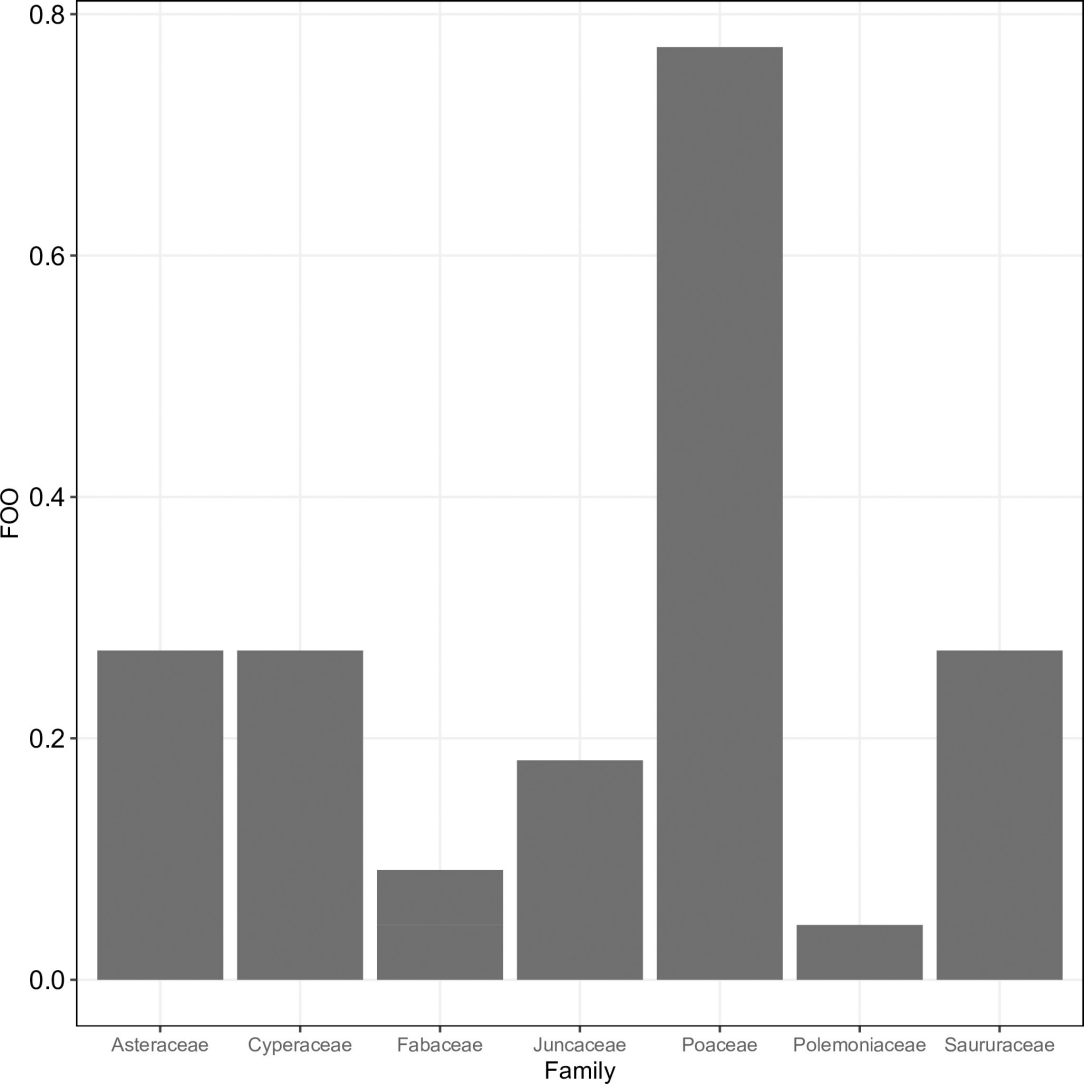
Frequency of occurrence (FOO, y-axis) of all plant families detected by metabarcoding (listed on x-axis) in fecal pellets of Amargosa voles.

When data from all samples were considered together, the family with the greatest frequency of occurrence by a considerable margin was Poaceae with the single saltgrass genus (almost 80%). Asteraceae (ragweed, sunflower (*Helianthus* sp.) and marsh aster (*Almutaster* sp.)), Cyperaceae with bulrush, and Saururaceae with yerba mansa were roughly equivalent, at approximately 25% (Fig 1). When the mean RRA was calculated for each marsh, saltgrass emerged as the most abundant non-bulrush diet item in all marshes except Marsh 9. In that marsh, mean RRA was higher for yerba mansa (mean RRA = 0.67), which was also abundant in Marsh 7 (mean RRA = 0.25). Other major contributors in some marshes included seaside arrowgrass in Marsh 21 (mean RRA = 0.17) and Marsh 54 (mean RRA = 0.31), and sunflower in Marsh 9 (mean RRA = 0.15) and Marsh 39 (mean RRA = 0.08). Other items detected in moderate abundance in a single marsh included ragweed in Marsh 7 (mean RRA = 0.21) and clover in Marsh 39 (mean RRA = 0.16). Marsh aster was detected in low abundance in Marsh 21 (mean RRA = 0.08) and Marsh 54 (mean RRA = 0.02). Other rarely detected genera included *Gilia* and *Acmispon*, each with mean RRA < 0.02 in Marsh 54.

**Fig 2:**
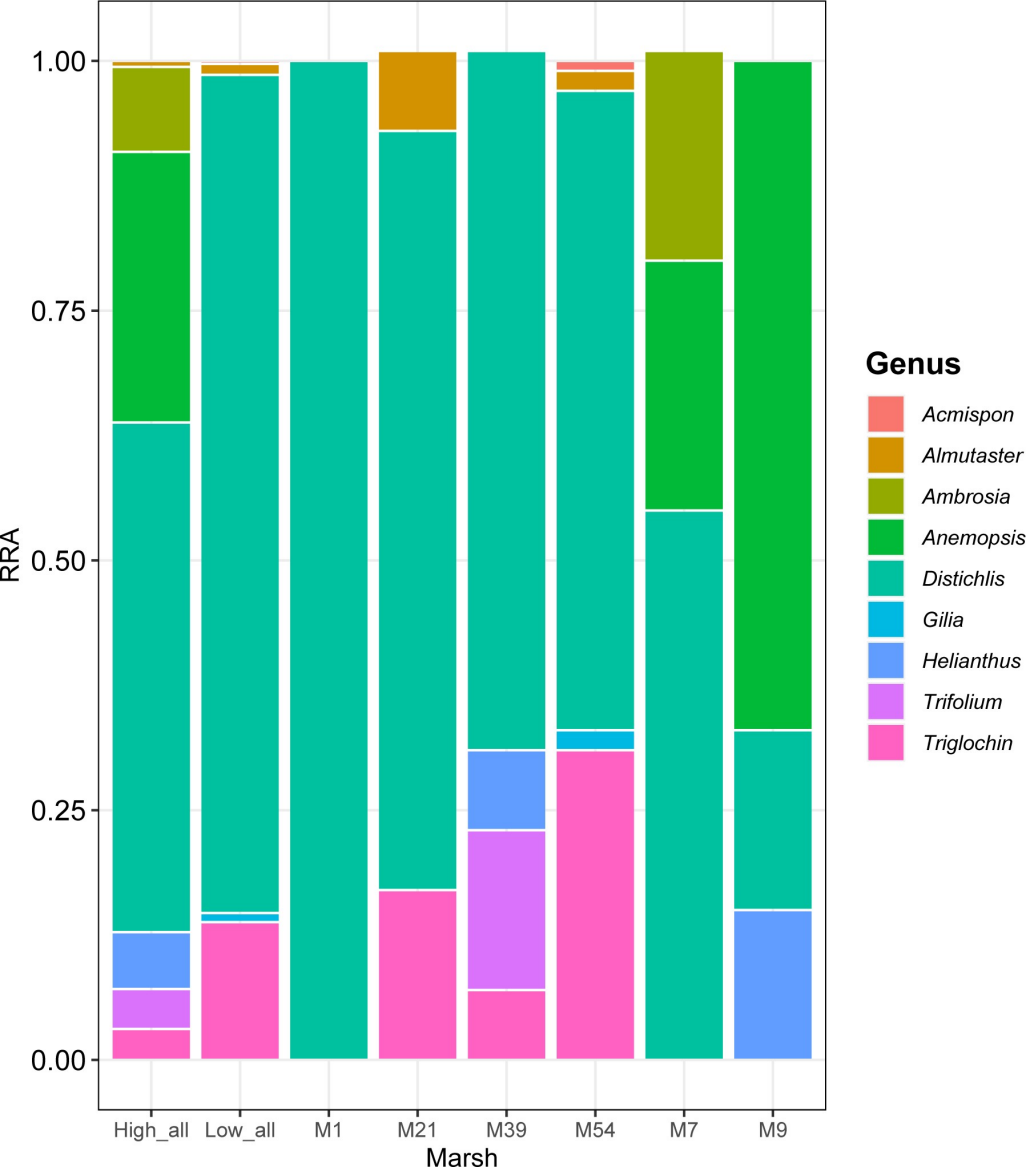
Relative read abundance (RRA, y-axis) of nine non-*Schoenoplectus* diet items detected by metabarcoding in feces of the Amargosa vole in six different marshes (listed on x-axis) near Tecopa, California, and when marshes were grouped as having high or low floral diversity (columns 1 and 2, respectively).

We compared diets from high and low plant diversity marshes (Fig 2). Eight of the ten plant genera detected by metabarcoding were found in diets of voles in high-diversity marshes: the only excluded genera were *Acmispon* and *Gilia*. There were five genera in diets from low-diversity marshes, where ragweed, yerba mansa, sunflower, and clover were not observed. A Student’s t-test did not indicate a significant difference in richness in diets between high and low-diversity marshes (P = 0.45). However, even though overall samples from high-diversity marshes indicated a relatively diverse diet, no single sample ever contained DNA associated with more than four genera of plant.

## Discussion

Accurate insight into the diet of an endangered habitat specialist is crucial to understanding its ecology, resource needs, and best practices for conservation. The Mojave Desert-endemic Amargosa vole has been described repeatedly as an obligate specialist on three-square bulrush. However, we demonstrated that this plant species lacks available dietary energy and protein to sustain a rodent, and that voles in the wild consume other plant species, based on remote-camera observation, microhistological analysis of feces, and DNA metabarcoding of feces. Although there were slightly more plant species represented in the feces of voles in high-diversity marshes, the finding that these differences were not significant is consistent with low statistical power but may also suggest that voles actively seek less abundant plants in their diet, likely because those plants confer greater nutritional value necessary for basal metabolic needs and other nutrient demands.

In marshes occupied by Amargosa voles, bulrush is the predominant canopy cover, most successful vole captures occur in bulrush, and voles that are monitored by radio-telemetry spend the majority of their time in bulrush [12, 13, 36]. Despite this habitat specialization, bulrush has inadequate total energy and protein to sustain voles [37]. Even though fat and protein concentrations are highest in bulrush seeds, microhistology analysis yielded primarily bulrush leaves, probably because seeds have very little mass. Calorically, bulrush leaves and seeds are comparable to tundra monocots, on which other vole species survive [20]. However, protein appears to be the critically limiting nutrient for voles. Although bulrush seeds contain the highest concentration of protein, a vole would need to consume 250g (dry weight) of seeds per day to meet its protein requirement, greater than three times the mean body mass of an adult Amargosa vole.

The assays we employed to detect diet constituents in vole feces could have been biased because samples were not directly collected from the stomach, and were only collected in spring. Moreover microhistology and metabarcoding vary in sensitivity and specificity. Systematic surveys of stomach contents of an endangered rodent are possible, but in one study, there was 7.7 times better detection of diet items in stomach than feces of rats (*Rattus* spp.), mice, and hedgehogs (*Erinaceus europaeus*, [38]). Due to variable sensitivity and specificity across assays, we could not comprehensively determine all plants consumed and the relative frequency of consumption. Microhistology has been used for a variety of mammals, including rodents such as the Mohave ground squirrel (*Xerospermophilus mohavensis*). However, quantitative assessment of diet is limited by differential digestibility of potential diet items. In addition, specificity of identification of cellular material varies based on the experience of the analyst. Advantages of this technique are that it can distinguish plant life stage and can identify some arthropods and fungi in addition to plants.

Our analyses should be interpreted cautiously because of detection bias in assays, such that quantifying diet using metabarcoding for example would be misguided. This is because it can over-represent rare taxa [39–43], and some specimens are not detected due to degradation during digestion [44]. PCR inhibition was deemed unlikely, because some targets amplified well, including multiple plant genera as well as our internal controls. Of concern was the limitation that the molecular assay under-represented the probable dietary contribution of bulrush. Several processes may contribute to taxonomic biases in a metabarcoding assay. Digestion may disproportionately degrade taxa, primer mismatches may inhibit amplification of particular taxa, or the availability of published reference data may limit assignment of ASVs [45]. In the present study the molecular assay appeared poorly suited to bulrush as evidenced by its 16% sensitivity in positive controls. Although this aided our ability to discover alternative food items by reducing the possibility of bulrush DNA dominating the reads, additional primer design and validation would be necessary in the future to more comprehensively catalog diet items. However, our ability to assess primer alignment was limited due to a dearth of public sequences that covered both forward and reverse binding sites. The two available ITS2 sequences of *S*. *americanus* (AF190621 and KC677959) perfectly matched the forward primer but did not have the reverse binding site available. The closest related species with reverse primer sites available either had perfect alignment (e.g., *S*. *tabernaemontani*; KF893304) or a single mismatch 12 bp from the 3’ end of the reverse primer (e.g., *S*. *pungens*; DQ385591). While this level of mismatch is unlikely to significantly inhibit amplification [46], it is possible *S*. *americanus* has unique additional mismatches in the primer-binding site that further reduced amplification. Future research on Amargosa vole diets could focus on generating a comprehensive sequence database of endemic plant populations (e.g., [47]) and developing tailored primer sets.

Metabarcoding also revealed plant items that were not hypothesized to be important to Amargosa voles. Specifically, four out of five marshes had DNA detections of plants that were not observed during vegetation surveys. In some cases these may have represented instances of voles foraging beyond marsh boundaries. For example, we detected *Acmispon* and clover which are not known to occur in Tecopa marshes. We also found *Gilia* which is a road-side weed; consuming these plants would require voles to leave the marsh to access them. In isolation, the metabarcoding dataset likely overrepresented the importance of these peripheral diet items. However, in tandem with microhistological data, metabarcoding provided a complementary and more diverse perspective of Amargosa vole foraging patterns. As has been previously demonstrated in herbivorous dietary studies [48, 49], use of multiple different technologies provided greater insight into Amargosa vole diet than any one alone.

While we present evidence that Amargosa voles consume other foods in addition to bulrush, voles do have physiological and anatomical gastrointestinal adaptations that increase their ability to use bulrush as a major or sole diet item. These include the presence of a high pH “esophageal pouch” in their proximal stomach and an enlarged cecum as occur in the Japanese field vole *(Microtus montebelli)* [50, 51]. The esophageal pouch serves as foregut and the enlarged cecum as hindgut. Both support anaerobic microbial flora capable of high efficiency fermentation [52]. A particularly important component of Amargosa vole microflora appears to be *Lactobacillus* which may ferment both simple sugars and lactic acid. *Lactobacillus* associates with increased nutrient extraction efficiency and weight gain on diets high in fiber and low in protein [53, 54]. Coprophagy, practiced by Amargosa voles [35] and other small mammals with hindgut fermentation, can also increase nutrient retention and acquisition of nutrients from the microbiota [55, 56].

Another means by which Amargosa voles may acquire protein is through consumption of arthropods, although there has been limited evidence of such in other California vole subspecies [17]. We have observed captive Amargosa voles in a breeding colony ingesting small crickets (Castle and Foley, unpub. data) and found microhistological evidence of insects in two fecal samples from wild voles. It is unknown how important these arthropods may be, as they were in very low numbers and may have been ingested inadvertently. Alternatively, different natural diets may be utilized in different seasons. For example, Antelope ground squirrels (*Ammospermophilus leucurus*) consumed the largest amount of arthropods when green vegetation was absent or scarce [57]. Given that we sampled vole feces during spring when marsh leaves and seeds are at their peak, it is possible that diet may differ and there may be more evidence of insects in samples collected in fall and winter months when resources are more sparse and Amargosa vole body mass tends to be low [13].

Diet requirements are critically important elements of the ecology of a species like the Amargosa vole which has been reported to be a habitat specialist, even though its conspecifics appear much less specific in their habitat and diet utilization. Better management of this endangered species and its habitat will be guided by clearer insights into its ecology. Even when samples were obtained from voles in marshes with low plant species diversity, there was strong evidence in both microhistological and metabarcoding data for a high variety of diet items. Voles may venture outside of their resident marshes to forage for food during which time they leave the protection of bulrush canopy and become more vulnerable to numerous, particularly avian, predators [11]. Habitat management for the Amargosa vole might be improved by including high plant species diversity, rather than aiming for bulrush monoculture. In the bigger picture, the growth of bulrush and other plants in the marshes is highly dependent on abundant and reliable water, which is affected by local and regional use, climate change, and drought to a greater extent than some other plant species. Seasonal and long-term trends could modify plant communities. If this occurs, it would be important to be able to predict the impacts of such modifications on voles, which would require that we understand whether voles might be able to burrow in ground for protection rather than in bulrush litter (although the ground in Tecopa outside marshes is hard-packed desert pavement and may be difficult to excavate), and utilize other plant species for diet.

Food availability and forage quality can affect individual physiology [5] and influence population dynamics [4] and inter- and intra-species interactions [3]. Diet analysis is a valuable tool for conservation management of endangered species including the Amargosa vole, the salt marsh harvest mouse (*Reithrodontomys raviventris*) [47], and the Pacific pocket mouse (*Perognathus longimembris pacificus*) [58]. Our results suggest that improved molecular tools are needed that are optimized for the plant communities in the central Mojave Desert, and that additional diet data should be collected from voles in disturbed habitat. Nevertheless, we clearly document flexible use of plant species in the diet of the Amargosa vole, and that such flexibility may be physiologically necessary due to inadequacy of bulrush to provide dietary energy and protein. Water depth and the presence of bulrush have been shown to be important determinants of persistence for the species [13, 59], and earlier workers have inferred that it is bulrush per se that is necessary for the voles, logically implying that restoration efforts should aim to reproduce bulrush monoculture which is characteristic of multiple marshes in the area. In contrast, this study highlights how better diet and natural history data can improve recommendations to recover endemic and endangered species. Our data would suggest that a more biodiverse ecosystem could actually provide greater benefit for recovery of this endangered species than pure bulrush, and that restoration be done to support available water, possible available soil for burrowing, and plant diversity.
